# Environmental Factors, Toxicants and Systemic Lupus Erythematosus

**DOI:** 10.3390/ijms150916043

**Published:** 2014-09-11

**Authors:** Anselm Mak, Sen Hee Tay

**Affiliations:** 1 Division of Rheumatology, Department of Medicine, University Medicine Cluster, National University Health System, 1E Kent Ridge Road, Level 10, NUHS Tower Block 119228, Singapore; E-Mail: sen_hee_tay@nuhs.edu.sg; 2 Department of Medicine, Yong Loo Lin School of Medicine, National University of Singapore, Singapore 119228, Singapore

**Keywords:** environmental factors, toxicants, genetics, epigenetics, T-cells, systemic lupus erythematosus

## Abstract

Systemic lupus erythematosus (SLE) is an immune-complex-mediated multi-systemic autoimmune condition of multifactorial etiology, which mainly affects young women. It is currently believed that the onset of SLE and lupus flares are triggered by various environmental factors in genetically susceptible individuals. Various environmental agents and toxicants, such as cigarette smoke, alcohol, occupationally- and non-occupationally-related chemicals, ultraviolet light, infections, sex hormones and certain medications and vaccines, have been implicated to induce SLE onset or flares in a number case series, case-control and population-based cohort studies and very few randomized controlled trials. Here, we will describe some of these recognized environmental lupus triggering and perpetuating factors and explain how these factors potentially bias the immune system towards autoimmunity through their interactions with genetic and epigenetic alterations. Further in-depth exploration of how potentially important environmental factors mechanistically interact with the immune system and the genome, which trigger the onset of SLE and lupus flares, will certainly be one of the plausible steps to prevent the onset and to decelerate the progress of the disease.

## 1. Introduction

Systemic lupus erythematosus (SLE) is a systemic autoimmune disease chiefly mediated by immune complexes that lead to inflammatory injury [1]. While the etiology of SLE is still not fully understood, the varied penetrance and lack of concordance of the disease amongst genetically identical twins suggest that both susceptible genetic profiles and environmental triggers are likely instrumental in inducing the onset of the disease and disease flares [2]. Over the past few decades, a number of environmental factors, such as cigarette smoke, alcohol, occupationally- and non-occupationally-related chemicals, ultraviolet light, hormones, infections, especially those caused by viruses, and vaccines, have been advocated to induce lupus and disease flares in a number of case series, case-control and population-based cohort studies and very few randomized controlled clinical trials [3]. In this review, we will describe and explain how, mechanistically, some of these recognized environmental factors may contribute to the onset and perpetuation of SLE through their interactions with epigenetic mechanisms in genetically susceptible individuals. However, infections, in particular those that their linked to SLE, have been described, such as the Epstein Barr virus and parvovirus, and will not be elaborated in this review. Although a cure for SLE will unlikely materialize in the very near future, further in-depth exploration of how important environmental factors mechanistically interact with the immune system and the genome to trigger the onset of SLE and disease flares will likely be one of the initial steps to prevent and decelerate the progress of the disease and enhance focused identification of therapeutic targets.

## 2. Potential Mechanisms of Induction of SLE-Interactions amongst Genetics, Epigenetics and Environmental Factors

### 2.1. Proposed Molecular Mechanisms

It is increasingly evident that various environmental triggers of SLE skew lupus CD4^+^ T-cells towards autoreactivity by altering the epigenetic mechanisms [4]. In brief, epigenetic mechanisms include DNA methylation and histone modifications, which involve methylation of the cytosine base of the DNA and deacetylation, ubiquination and trimethylation of histone tails, respectively [5]. Generally, these epigenetic mechanisms inhibit gene transcription, with subsequent silencing of the expression of certain unnecessary and even harmful genes [6]. In fact, during each cycle of mitosis, DNA methyltransferase (mainly Dnmt1 as the maintenance enzyme), the enzyme that catalyzes and inherits DNA methylation to the daughter cell, is activated [7]. Deficiency and inhibition of Dnmt1 have been shown to enhance the expression of methylation-sensitive genes, which are designated to be silenced. For instance, by inhibiting Dnmt1 upregulation during mitosis or by depleting mitotic cells of methyl donors, normally silenced genes in certain T-cell subsets, such as those encoding perforin and the killer cell immunoglobulin-like receptor family, interleukin (IL)-4, -5 and -13 and interferon (IFN), are abnormally overexpressed in CD4^+^, Th1 and Th2 cells, respectively, in addition to overexpression of CD70 and CD40L in these cells [8,9,10].

**Figure 1:**
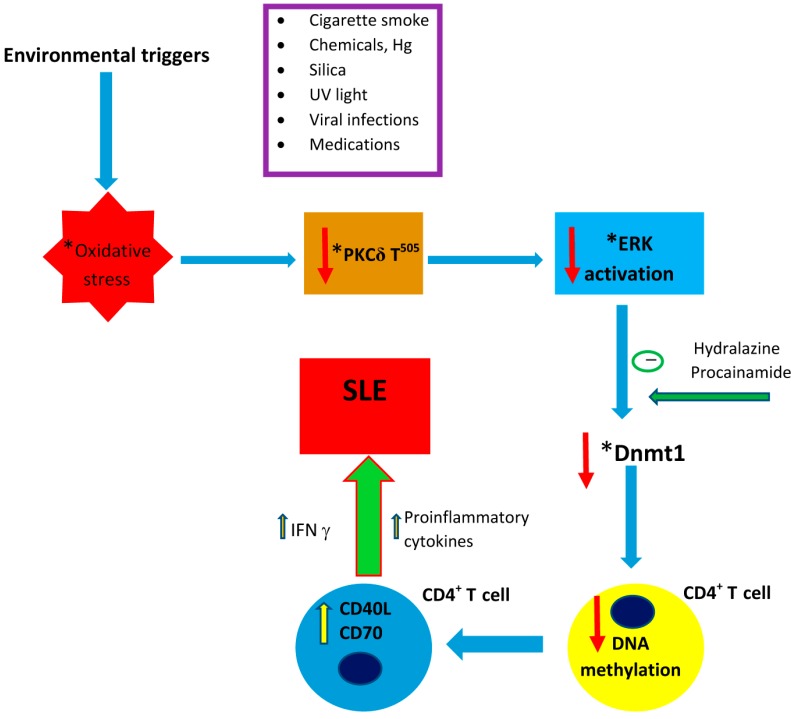
Schematic presentation of the proposed pathways linking environmental factors to the development of systemic lupus erythematosus (SLE). See the text for details. * Denoted outside the cell in this diagram for the sake of the clarity of presentation. These processes indeed take place inside the CD4^+^ T-cell; Hg, mercury; UV, ultraviolent; PKCδ T^505^, phosphorylated protein kinase C δ; ERK, extracellular signal-regulated kinase; Dnmt1, DNA methyltransferase 1; IFN γ, interferon γ; and SLE, systemic lupus erythematosus.

In patients with SLE, the DNA of their CD4^+^ T-cells is hypomethylated, and their T-cells tend to be autoreactive in response to self-class MHC II molecules without strengthening of signals from autoantigens [7,11]. During mitosis, the level of Dnmt1 is regulated partly by the activated extracellular signal-regulated kinase (ERK) signaling pathway [12]. ERK activity has been found to be suppressed in lupus CD4^+^ T-cells [12], and recent studies have demonstrated that the lupus ERK signaling defect is indeed related to impaired protein kinase C (PKC) phosphorylation [13]. On further analysis of 16 lupus patients with a range of disease activity, oxidative-stress-induced peroxynitrite (ONOO^−^) in lupus CD4^+^ T-cells was found to contribute to site-specific decrease in phosphorylation of PKCδ T^505^, leading to functional loss of PKC and parallel reduction of ERK phosphorylation, which were significantly correlated with higher lupus disease activity [14]. As a result of reduced ERK activity in lupus CD4^+^ T-cells, expression of Dnmt 1 is reduced, DNA hypomethylation ensues and the CD4^+^ T-cells become autoreactive (see Figure 1 for the proposed molecular pathways linking oxidative stress, suppression of the PKC and ERK pathways and reduction of Dnmt1 with lupus development).

### 2.2. Evidence from Animal Models

Neither the sole presence of lupus-susceptible genes nor the exposure of lupus-triggering environmental factors alone is sufficient to trigger SLE. Knowledge gained from animal models strongly suggests that the presence of environmental, genetic and epigenetic factors are necessary to induce lupus. An example is the development of anti-dsDNA antibodies and an interferon (IFN) signature of leukocytes without tissue damage in transgenic C57Bl/6 mice, which express a dominant negative ERK in T-cells when they were fed with doxycycline in water [15]. Intriguingly, when C57Bl/6 mice were crossed with lupus-prone SJL mice, which carry lupus-susceptible genes, the resultant C57Bl/6xSJL mice developed immune-complex-mediated glomerulonephritis besides lupus serology and the IFN signature [4]. This illustrates that while DNA demethylation of T-cells alone is sufficient to induce anti-dsDNA antibody formation and an IFN signature, the presence of lupus-susceptible genes is necessary to drive tissue and end-organ diseases. In another experiment, only ovariectomized female B6xSJL lupus-prone mice developed anti-dsDNA antibodies and glomerular IgG depositions in kidneys, but not in male transgenic mice when they were fed with doxycycline [16]. Hence, the presence and hypomethylation of both X chromosomes are crucial to engender CD40L overexpression in the female mice, which induced lupus-like disease [16]. Besides genetically engineered murine models, which develop lupus-like disease spontaneously, lupus can be readily induced in wild-type mice by administration of chemicals. The use of 2,6,10,14-tetramethylpentadecane (pristane) is one of the most recognized methods to induce lupus-like disease in the murine systems [17,18]. With a single injection of pristine, mice of certain genetic backgrounds, including those of Balb/c and SJL, develop lupus-like disease characterized serologically by the development of autoantibodies, such as anti-histone IgM, anti-Sm IgG and anti-ribosomal P IgG [19]. Albeit a well-recognized chemical-induced murine lupus model, researchers should realize that the disease manifestation of immune-complex disease differs when mice of different genetic backgrounds are chemically induced. For instance, mice of the SJL and New Zealand White/Black (NZW/B) backgrounds tend to manifest glomerulonephritis after pristine injection, while those of the DBA1 background develop arthritis and an IFN signature [20]. Thus, while these facts illustrate the importance of the appropriate choice of mouse models to answer specific research question and identify drug targets, these also substantiate the importance of genetics and environmental factors in triggering the disease, which has a protean clinical manifestation.

## 3. Environmental Agents Data from Observational Studies

A number of environmental agents, such as cigarette smoke, viral infection and various chemicals, has been demonstrated to induce oxidative stress [15], and oxidative stress has been shown to inhibit and/or reduce Dnmt1 level, which subsequently reduces DNA methylation in CD4^+^ T-cells and enhances autoimmunity [15].

### 3.1. Ultraviolent Light

Ultraviolet (UV) light, in particular UV-A1 and UV-B, can induce disease flares in patients with SLE and trigger disease onset of SLE [21]. Furthermore, the ability of UV light to induce SLE or lupus flares appears to be dose dependent [22]. It is evident that UV-B induces apoptosis of keratinocytes and other dermal cells, and such progress releases a large amount of autoantigens and pro-inflammatory cytokines to the circulation, triggering autoimmune-related systemic inflammation [22]. In low doses of UV-B, normal cascade-dependent apoptosis has been induced in keratinocytes, while in moderate and high doses, DNA fragmentation, increase in IL-1α expression and necrosis of keratinocytes have been observed [22]. In addition, *in vitro* study found that instead of the translocation of the immunogenic Ku, Sm and DNA antigens onto the cell membrane, high-dose UV-B has been shown to lead to the release of these autoantigens into the cytoplasm and even to the supernatants when the cellular integrity was compromised [22]. Taken together, intermediate- and high-dose UV-B exposures promote pro-inflammatory apoptosis and necrosis, accompanied by the release of autoantigens and huge amount of pro-inflammatory cytokines, which trigger inflammatory response [22].

### 3.2. Vitamin D Deficiency

Due to advice for avoidance of UV light, a potential problem in SLE patients is vitamin D deficiency [23]. Beyond calcium and phosphate metabolism and maintenance of normal bone physiology, the protective role of vitamin D in modulating the immune system has been increasingly recognized [24]. In fact, deficiency of vitamin D was demonstrated to be associated with higher lupus disease activity [25]. In a recent study, a 20-ng/mL increase in serum 25-OH vitamin D was shown to be associated with a 15% decrease in the odds of clinically-important proteinuria (urine protein-to-creatinine ratio > 0.5) in patients with SLE [25]. Using chromatin immunoprecipitation followed by massively parallel DNA sequencing (ChIP-seq), 2776 binding sites for vitamin D receptor binding were found along the length of the human genome. Significantly, there was significant enrichment of these binding sites in regions associated with active chromatin, including DNAase I-hypersensitive sites and specific histone modifications [26]. These results strongly imply that vitamin D, upon binding to its cognate nuclear receptor, exhibits a potential regulatory role in gene expression important for SLE pathogenesis and modulation of disease activity. Indeed, the importance of vitamin D in regulating innate and adaptive immunity has been highlighted by studies demonstrating inhibition of interferon α (IFN α)-mediated monocyte differentiation into dendritic cells, increased serum IFN α activity in SLE patients with vitamin D deficiency, decreased proliferation and production of polyclonal immunoglobulin G (IgG) and anti-dsDNA IgG in SLE PBMC treated with vitamin D and the regulation of dividing plasmablasts and long-lived plasma cells [27,28,29,30]. The regulatory role of vitamin D in the downstream production of anti-dsDNA IgG may herald the potential relationship of vitamin D and SLE renal disease [31]. Further, the loss of vitamin D binding protein (and protein-bound vitamin D metabolites) in the urine may reflect the magnitude of proteinuria exhibited by SLE patients with renal disease, resulting in low vitamin D levels [32].

### 3.3. Smoking

Many toxic substances that activate alveolar macrophages, induce myeloperoxidase activity and production of free radicals are found in tobacco smoke [33]. While a few case-control studies have found that previous and current smoking were associated with the risk of SLE and discoid lupus [34], and current smoking appeared to be a stronger risk factor for SLE than past smoking, a meta-analysis of two cohort and seven prevalent studies found a very weak association (odds ratio (OR) 1.5, 95% confidence interval (95% CI): 1.09–2.08) for the development of SLE in current smoking *vs.* never smokers [35]. There is no increase in risk of SLE as ex-smokers as compared with never-smokers [35]. Interestingly, after adjustment for alcohol consumption and socioeconomic status, which confound smoking status, the ORs augmented to 2.07 (95% CI: 1.33–3.23) and 1.76 (95% CI: 1.09–2.83), respectively, indicating that smoking *per se* was able to increase the risk of SLE [35]. Thereafter, three large population-based cohorts in the U.S. failed to demonstrate smoking and the development of SLE [36]. In Asia, a case-control study of 171 female lupus patients and 492 female healthy subjects in Japan revealed an OR of 3.06 (95% CI: 1.86–5.03) of the occurrence SLE in current smokers [37]. Conducted by the same group of Japanese investigators, it was found that the presence of at least one G allele of TNFRSF1B *rs1061622* conferred an increased risk of SLE (OR 1.56, 95% CI: 0.99–2.47). The attributable proportion due to the interaction between the TNFRSF1B *rs1061622* genotypes and smoking was estimated to be 0.49 (95% CI: 0.07–0.92), suggesting that 49% of the excess risk for SLE in smokers with at least one G allele was due to an additive interaction [38]. In the profile of Kyushu Sapporo SLE (KYSS) study, which was also conducted in Japan, a combination of current and former smoking was associated with higher odds for the occurrence of SLE in one region (Kyushu in southern Japan), but not in the other (Hokkaido in northern Japan), after adjustment for age and alcohol drinking (one drink/week) [39]. However, in contrast to studies performed in North America, a dose-response relationship between smoking and the risk of SLE has been established in the two Japanese studies [39]. In addition to the risk of development of SLE, smoking was noted to be associated with skin flares in patients with SLE [34]. It is likely that tobacco smoke may have reduced the efficacy of antimalarials, which eventually induces exacerbation of cutaneous lupus [40].

### 3.4. Alcohol

No clear association has yet been convincingly reported with respect to the potential risk of the development of SLE and alcohol consumption, especially since the habit of smoking and alcohol intake often coexist, which confounds interpretation [39,41]. In the KYSS study, while light or moderate alcohol consumption did not appear to increase the risk of SLE significantly, heavy alcohol consumption (>4–5 days/week) was shown to be associated with odds of 4.49 (95% CI: 1.43–14.08) for the occurrence of SLE after adjustment for age in the people of Kyushu, but not in those of Hokkaido [39]. In a subsequent study performed by Kiyohara *et al*. in Kyushu using never drinkers as the reference, it was found that light and moderate alcohol consumption indeed conferred a 62% significant reduction of risk in the occurrence of SLE [37]. Being different in North America, the prospective Black Women’s Health study demonstrated that neither past nor current alcohol consumption was associated with the development of SLE [42]. In an Internet-based study of 114 lupus cases and 228 matched controls, current drinking (>2 drinks/week and >2 drinks/day) was inversely associated with the development of SLE [43]. However, drinking prior to lupus development is not associated with subsequent occurrence of SLE, and people diagnosed to have SLE were more likely to quit drinking, although it did not reach statistical significance [43]. Finally, in a meta-analysis of six prevalent studies, a significant protective effect of moderate alcohol consumption against SLE was found (OR 0.72, 95% CI: 0.547–0.954) in those lupus patients who were treated for SLE for less than 10 years [44].

### 3.5. Occupationally- and Non-Occupationally-Related Chemicals

People working in the rural farming industry and sandblasting may be exposed excessively to crystalline silica. Silica is an adjuvant that can induce the production of IL-1 and tumor necrosis factor α (TNF α). The Carolina Lupus Study suggested that crystalline silica did confer a risk of development of SLE [45], and the results were replicated by two subsequent studies [46,47]. In the most recent study, which reviewed over 1000 patients in North America who developed SLE for over 20 years, the prevalence of SLE related to silica exposure was 0.1/100, with a relative risk (RR) of 2.53 (95% CI: 0.30–21.64) compared with the general population [48]. In Asia, the contamination of rice oil with chlorinated compounds polychlorinated biphenyls/dibenzofurans (PCBs/PCDFs) in Taiwan in 1979 revealed an excessive frequency of lupus development amongst the exposure group after a 24-year period, in addition to death related to liver disease [49]. Occupational exposures to mercury (Hg), liquid solvents and pesticides have been demonstrated to increase the likelihood for SLE development [50].

Hg is a ubiquitous toxicant, for which its organic form, which is methylated, is mainly from contaminated seafood, while its inorganic form is chiefly found in dental amalgams and old-fashioned thermometers. Both organic and elementary Hg can induce anti-nuclear antibody (ANA) in murine models, as well as in humans [51,52]. Both methyl-Hg and inorganic Hg induce oxidative stress of T-cells by depleting thiol-containing antioxidants and glutathione, leading to the production of reactive oxygen species and enhancement of apoptosis and inactivation of PKCδ of CD4^+^ T-cells [53,54]. Occupation exposure of Hg was reported to increase the odds of developing SLE (OR 3.6, 95% CI: 1.3–10.0), and amongst the dental professionals, the OR of SLE development based on analyses of self-reported Hg exposure is 7.1 (95% CI: 2.2–23.4) [55].

Interestingly, the use of lipstick and hair dye have been reported to induce onset of SLE. Lipstick, which contains a variety of chemical compounds, such as eosin and phthalates, has been shown to induce photosensitivity and lupus flares, as well as the production of anti-dsDNA antibodies and progression of renal disease in NZB/W F1 mice, respectively, partly and potentially due to the breach of immunological tolerance by molecular mimicry [56]. In an Internet-based case-control study, the use of lipstick at least thrice weekly was found to be associated with the occurrence of SLE, after adjustment for age, hair dye use and alcohol consumption, with an OR of 1.71 (95% CI: 1.04–2.82) [57]. More frequent use of lipstick (seven days weekly) and early use of lipstick (before the age of 16) were associated with even marginally higher risks for the development of lupus, with ORs of 1.75 (95% CI: 0.89–3.44) and 1.95 (95% CI: 1.01–3.76), respectively [57]. While the risk of induction of lupus by hair dye treatment, which contains aromatic amines, is theoretically present, it is largely refuted by large observational studies in human lupus. Mechanistically, the potential lupus-inducing effect of aromatic amines is related to the fact that the amines are metabolized by acetylation, a process that shares a similar pathway as that of hydralazine [58]. Thus, amongst slow acetylators, the accumulation of aromatic amines might be able to induce SLE in genetically susceptible individuals (see Section 5. Medications for details). In a cross-sectional study performed in South and North Carolina, which involved 265 lupus patients and 355 healthy controls, the use of hair dye in women was shown to be associated with a small, yet marginally significant risk of SLE (OR 1.5, 95% CI: 1.0–2.2). In addition, use of hair dye for six years or more appeared to increase the risk (OR 1.7, 95% CI: 1.0–2.7) [59]. However, in a prospective study in Spain, which followed 91 lupus patients and 22 patients with cutaneous lupus for 12 years, the use of hair dye treatment was not associated with disease flares and damage accrual in both groups [60].

## 4. Vaccinations

The presence of adjuvants in the vaccines may be a potential reason why triggering of SLE has been believed to be linked to vaccinations [61]. Although the theoretical potential of triggering of lupus flares by vaccinations has been advocated [62,63], large case-control and prospective studies have revealed no evidence of exacerbation of existing SLE and the triggering of the onset of SLE in the most commonly prescribed vaccines, including influenza, hepatitis B, human papilloma virus and varicella vaccines [64,65,66].

## 5. Medications

Certain medications can induce lupus-like disease. For example, procainamide, a Class 1a anti-arrhythmic drug, can induce lupus-like disease in susceptible individuals by behaving as a competitive inhibitor of Dnmt1 [67]. Hydralazine, an anti-hypertensive agent, as well as an anti-heart failure agent, when used in combination with nitrates reduces Dnmt1mRNA and protein levels in T-cells by deactivating the ERK pathway [68]. In the contemporary era of biologic therapy, anti-TNF has been increasingly used in the treatment of inflammatory arthritis, but cases of anti-TNF-induced lupus have been reported [69]. Interestingly, in contrast to lupus-like syndrome induced by hydralazine and procainamide, the occurrence of anti-dsDNA antibodies and hypocomplementemia is more common in anti-TNF-induced lupus, although the mechanism is not fully understood [3]. Indeed, one of the main distinguishing features between drug-induced and idiopathic lupus is the absence of end-organ inflammation, such as glomerulonephritis in the former, although arthritis and serositis are not uncommon in patients with drug-induced lupus. While the pathogenic mechanisms of idiopathic and drug-induced lupus are believed to be different, withdrawal of offending medications often completely alleviates the symptoms of drug-induced lupus. Nevertheless, drugs that are known to induce lupus are generally not recommended in patients with SLE.

In general, estrogens are immunostimulatory in that they induce polyclonal expansion and proliferation of B-cells in physiological doses [70,71]. Recent data suggest that the role of estrogens in the pathogenesis in lupus is potentially complex and age dependent. In the NZW/B F1 model, deficiency of the estrogen receptor-α was shown to have a reduction of the production of anti-histone and anti-dsDNA IgG, accompanied by attenuating glomerulonephritis and increasing survival, both in female and male mice [72]. However, repletion of estrogens in ovariectomized adult NZW/B F1 mice reduced albuminuria and did not appear to increase the progress of lupus in these mice [73]. Additionally, estrogens enhance the production of calcineurin and subsequent upregulation of CD40L on T-cells [74]. At a supraphysiological dose, estrogens are able to inhibit IL-2 production. Thus, they theoretically induce the higher susceptibility of SLE in females of reproductive age or supplement, because of inhibition of Th1 responses, CD40L upregulation and bias towards Th2 responses [70,75]. In clinical practice, a number of case series described the induction of SLE and disease flares in SLE patients who took combined oral contraceptive pills, but a randomized controlled trial found that combined oral contraceptives did not confer a higher risk of disease flares in women with clinically stable SLE [76]. On the other hand, another randomized controlled trial did find a higher risk of mild to moderate lupus flares in postmenopausal women with SLE who used hormone replacement therapy [77].

## 6. Conclusions and Future Directions

A number of environmental agents that have been shown to be associated with the occurrence of SLE were described. Nevertheless, data are mainly based on case-control studies, which are undoubtedly limited by recall bias, which fails to establish a causative effect. Large and multi-center prospective studies are required to address the cause-effect relationship, as well as to confirm and refute data from cross-sectional studies, especially with regard to the potential protective effect of mild to moderate alcohol consumption against SLE. As for meta-analyses, while combining data of different studies can increase the power to detect differences, heterogeneity in population, data collection, definition of disease and statistical methods will weaken the overall validity of the effect size. A more convincing method to enhance the value of meta-analyses is the use of the Bayesian method for data combination based on posterior probability [78].

Further elucidation of the molecular mechanisms and pathways involved in the abilities of various environment agents of interest to induce SLE is important, because this helps accelerate the identification of potential treatment targets for the disease. Currently, although CD4^+^ T-cells have been relatively well studied for their relationship between oxidative stress, hypomethylation and autoreactivity, other cell types, such as lupus B-cells and renal tubular cells, may be pathologically important, because intracellular evidence of oxidative stress has long been convincingly demonstrated in these cells [79,80,81].
